# Effect of Aerobic Exercise Training on Chinese Population with Mild to Moderate Depression in Hong Kong

**DOI:** 10.1155/2014/627376

**Published:** 2014-03-30

**Authors:** Cassandra W. H. Ho, S. C. Chan, J. S. Wong, W. T. Cheung, Dicky W. S. Chung, Titanic F. O. Lau

**Affiliations:** ^1^Physiotherapy Department, Tai Po Hospital, Wing E, Ground Floor, Tai Po, New Territories, Hong Kong; ^2^Department of Psychiatry, Tai Po Hospital, Tai Po, New Territories, Hong Kong

## Abstract

*Background*. Exercise has been suggested to be a viable treatment for depression. This study investigates the effect of supervised aerobic exercise training on depressive symptoms and physical performance among Chinese patients with mild to moderate depression in early in-patient phase. *Methods*. A randomized repeated measure and assessor-blinded study design was used. Subjects in aerobic exercise group received 30 minutes of aerobic training, five days a week for 3 weeks. Depressive symptoms (MADRS and C-BDI) and domains in physical performance were assessed at baseline and program end. *Results*. Subjects in aerobic exercise group showed a more significant reduction in depressive scores (MADRS) as compared to control (between-group mean difference = 10.08 ± 9.41; *P* = 0.026) after 3 weeks training. The exercise group also demonstrated a significant improvement in flexibility (between-group mean difference = 4.4 ± 6.13; *P* = 0.02). *Limitations*. There was lack of longitudinal followup to examine the long-term effect of aerobic exercise on patients with depression. *Conclusions*. Aerobic exercise in addition to pharmacological intervention can have a synergistic effect in reducing depressive symptoms and increasing flexibility among Chinese population with mild to moderate depression. Early introduction of exercise training in in-patient phase can help to bridge the gap of therapeutic latency of antidepressants during its nonresponse period.

## 1. Introduction

Depression is currently the third leading cause of global disease burden in 2004; it is estimated that it will become the first most common disabling disease by year 2030 [1]. With increasing tightness in healthcare budgets, there has been great interest in the development and evaluation of alternative or augmentation therapies for depression.

Depression is traditionally treated with pharmacological intervention or psychotherapy or a combination of both. However, the effect of treatment is often suboptimal. Despite new development of antidepressants for depression, at least 30% of depressed patients fail to achieve a satisfactory response [2]. Unwanted side effects induced by antidepressants may impair patient's quality of life and reduce compliance [3]. In up to 50% of acute depressive cases, antidepressants require 1 to 4 weeks before showing any therapeutic effect [4].

In recent years, physical activity [5] and exercise therapy have been extensively examined as treatment for depression, both as monotherapy and as augmentation to antidepressants [6]. Results from meta-analyses of exercise for depression have revealed an inverse relationship between exercise and depression [7] while others comment that exercise is as effective as cognitive behavioral therapy [7, 8]. Continuing to exercise following participation also lowers risk of relapse for patients diagnosed with major depressive disorder (MDD) [9].

When comparing aerobic training to no treatment [10] and resistance training versus standard treatment [11], both are equally effective in reducing depressive symptoms. Researchers found the positive effect of exercise training to be intensity related. A moderate exercise dose appears to have greater therapeutic effect for patients with depression when compared with a low exercise dose in adults with MDD of mild to moderate severity [10]. Chu et al. [12] further supported that subjects undergoing high intensity aerobic training reported significantly fewer depressive symptoms than those in low intensity group and stretching group in sedentary women with mild to moderate depressive symptoms.

Depression may present with feeling of tiredness and reduced motivation for physical activity, which ultimately leads to deconditioning and lower physical performance. Depressed mood is associated with increased risk of strength decline leading to disability [13], while handgrip strength has often been used as predictor of functional disability [14]. The behavioral attributes of depressed mood include physical inactivity may worsen the functional mobility which may cause muscle strength decline. Besides, individuals with MDD would experience changes in posture, like increased head flexion and increased thoracic kyphosis, and mild dissatisfaction with their body image during episodes of depression [15]. These changes would further reinforce depressive patients to become physically inactive leading to muscle strength decline. Aerobic exercise produces positive effect on physical performance thus breaking the vicious cycle and subsequently reduces depressive symptoms.

Early introduction of aerobic exercise training during hospitalization may help to supplement the therapeutic latency of antidepressants [16]. Up till now, there has been no reported study on effect of aerobic exercise training among Chinese individuals with depression. Therefore, there is a need to investigate the effect of aerobic exercise training in early in-patient phase among Chinese individuals with depression in local (Hong Kong) setting. This study aimed to assess the effect of a 3-week in-patient aerobic exercise training in addition to pharmacological intervention on depressive symptoms and physical performance among Chinese subjects with mild to moderate depression.

## 2. Methods

### 2.1. Study Design

A randomized, repeated measure, and assessor-blinded design was used.

### 2.2. Subjects

Between February 2010 and December 2011, all patients who were admitted to the Psychiatric Unit in Tai Po Hospital were initially screened using 21-item Chinese version of the Beck Depression Inventory (C-BDI). Subjects obtaining a C-BDI score of 9 or above and meeting the International Classification of Disease (ICD-10) criteria for MDD were recruited. Eligibility criteria included (1) males or females aged 18–64 years; (2) sedentariness, as defined by the absence of involvement in any structured or regular exercise activities over the past one month; (3) currently taking an antidepressant. Exclusion criteria were (1) unstable cardiopulmonary disease; (2) major orthopedic or neurological disease that limits exercise capacity; (3) malignancies in the past 5 years; (4) presence of another primary psychiatric disorder; (5) presence of alcohol or substance dependence within the past 6 months; (6) currently receiving psychotherapy or electroconvulsive therapy; (7) high suicide risk.

The objective of the study, possible risks, potential benefits involved, and the contents of the tests were explained to each subject prior to the study. All subjects were given a participant information sheet and written consent was obtained. The study procedures were approved by the Joint Chinese University of Hong Kong and New Territories East Cluster (CUHK-NTEC) Clinical Research Ethics Committee.

### 2.3. Sample Size Estimation and Randomization

Based on the meta-analysis by Lawlor and Hopker [7] with weighted mean difference in the Beck Depression Inventory −7.3 when comparing exercise group with no treatment, we calculated the estimated overall sample size of 52 (26 participants in each arm) achieving an 80% power at a two-sided 0.05 significant level.

Subjects were randomly allocated using blocked randomization into one of two groups: aerobic exercise group and control group. A random number list was generated by an investigator with no clinical involvement in the study using online research randomizer.

### 2.4. Study Protocol

A physiotherapist who was independent of the recruitment process for allocation consignment would interview all subjects, who were eligible for the study, using the Chinese version of Physical Activity Readiness Questionnaire (PAR-Q) by the Canadian Society for Exercise Physiology as a preparticipation screening. Individuals who had risk factors identified by the PAR-Q should get medical clearance before they participated in the aerobic exercise program. Before participation, a standardized introduction session was delivered to both the aerobic exercise group and control group.

#### 2.4.1. Aerobic Exercise Group

All subjects in aerobic exercise group attended 5 supervised exercise sessions per week for 3 consecutive weeks. An interval-training exercise regimen was adopted which included both upper extremity and lower extremity aerobic exercise training. Before each interval training, subjects would undergo a 5-minute warm-up with stretching exercise of large muscle group, followed by a 30-minute interval training at an intensity that maintained heart rate within the targeted training zone, and finally concluded with a similar 5-minute cooldown exercise (stretching exercise of large muscle group).

During the 30-minute interval training, subjects were instructed to exercise 3 bouts of 5-minute workout with prescribed training intensity at 40–59% heart rate reserve (HRR) or with modified rate of perceived exertion (RPE) of 4–6 which is a moderate intensity as according to the guideline of the American College of Sports Medicine [17]. Immediately after each 5-minute workout, participants exercised at a reduced intensity of 20–39% HRR or modified RPE of 2-3 for 5 minutes, all together making up a total of 30 minutes of aerobic interval training. Heart rate and RPE were monitored and recorded during each training session by a physiotherapist. In order to enhance the adherence of exercise training, all subjects were requested to record the amount of exercise in an exercise log book.

#### 2.4.2. Control Group

Subjects in control group were reminded to maintain their physical activity level as usual. A 10-minute stretching exercise on large muscle group was given for standardization.

### 2.5. Outcome Measures

Depression severity rated by clinician using the Montgomery-Asberg Depression Rating Scale (MADRS) was the primary outcome; all other outcomes were secondary. All the outcomes were assessed before and after the program. Demographic data, including age, gender, weight, height, and body mass index, were recorded at baseline to characterize the participants.

#### 2.5.1. Montgomery-Asberg Depression Rating Scale (MADRS)

MADRS is a 10-item scale used to measure the severity of depressive symptoms originally designed to be sensitive to the effects of antidepressant medications [18]. A score greater than 35 indicates severe depression, while a final score of 10 or below indicates remission [19]. MADRS has high internal consistency given the high correlation between all test items (*r* = 0.95) [20], interrater reliability ranging from 0.76 to 0.95 [21], and high correlation with the Hamilton Depression Rating Scale (between 0.80 and 0.90) [22]. All subjects were assessed by one of three assessors (2 psychiatrists and one trained physiotherapist) on the Montgomery-Asberg Depression Rating Scale (MADRS), who were blinded to the subjects' allocation throughout the study. In the present study, the interrater reliability among the 3 assessors was established (ICC = 0.95, *n* = 10).

#### 2.5.2. Chinese Version of the Beck Depression Inventory (C-BDI)

The Chinese version of the Beck Depression Inventory (C-BDI) is a 21-item self-rating instrument used to measure severity of depression [23]. The scale ranged from 0 to 63; scores between 10 and 18 indicate mild depression, between 19 and 29 indicate moderate depression, and between 30 and 63 indicate severe depression. The C-BDI was found to have good internal consistency (Cronbach's alpha = 0.846) and moderate concurrent validity (*r* = 0.566) when compared with the Chinese version of the Hamilton Depression Rating Scale [23]. It also yielded good sensitivity and acceptable specificity in assessing psychiatric patients with mixed diagnoses [24].

#### 2.5.3. Sit-and-Reach Flexibility Test

Low back and hamstring flexibility are one of the important components of physical performance [25] which strongly affect the posture and body image of a person. A standardized piece of equipment, the specially constructed sit-and-reach box, was used. Subjects sat on the floor with shoes off and placed the bottom of feet against the box with knees straight. Subjects reached forward along the measuring line with the palms facing downwards as far as possible [26]. The test procedure was repeated 3 times and the mean value was recorded. For easy analyses and comparing results, the zero mark was adjusted 23 cm (9 inches) before the feet.

#### 2.5.4. Handgrip Test

Handgrip strength has shown to be predictive of functional limitations and disability [14] and was classically measured by a handheld dynamometer. Subjects were asked to maximally squeeze the handheld dynamometer (JAMAR); test was repeated on both hands 3 times each and mean value was taken.

### 2.6. Statistical Analysis

For baseline demographics, descriptive statistics with frequency count, percentages, and mean ± standard deviation (SD) would be reported. Intention-to-treat analysis of all randomized subjects was conducted. Missing data were computed by carrying forward the last recorded observation. For the baseline demographics and clinical characteristics, cross tabulations (Chi-squared test or Fisher's exact test, when appropriate) and *t*-test were used for discrete and continuous variables. Between-group differences comparing mean change and the main effect of treatment were examined using *t*-test. All data were analyzed using SPSS 16.0 for Windows. All the statistic tests were two-tailed with significant level at *α* = 0.05.

## 3. Results

### 3.1. Participants Characteristics

**Figure 1:**
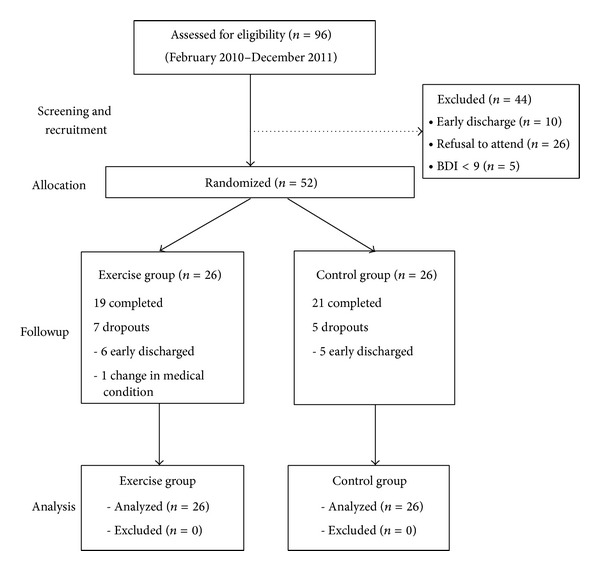
CONSORT diagram showing the randomization and subjects flow of the study.

Between February 2010 and December 2011, 96 individuals, admitted to the Psychiatric Unit of Tai Po Hospital (TPH), were screened for eligibility. 44 individuals were excluded from the study; reasons for ineligibility were early discharge from hospital (*n* = 10), BDI score less than 9 (*n* = 5), refusal to join the study (*n* = 26), and having physical problem that limits their exercise capacity (*n* = 3). Of those individuals who met the inclusion criteria, 26 were randomly assigned to exercise group and 26 were to control group. At the end, 40 participants completed the whole program; 12 participants (exercise group = 7, control group = 5) were unable to finish the 3-week training. No adverse side effect was reported in all participants. The major reasons for dropping out were early discharge (*n* = 11) except one who had a change in medical condition (*n* = 1) (Figure 1).

Baseline characteristics of the 52 subjects are shown in Table 1. There were no significant group differences in their demographic characteristics in age, sex, and body mass index. There was also no group significant difference in the 2 depression scores (MADRS and BDI) and physical performance (handgrip strength and sit-and-reach distance) at baseline (Table 1). The mean MADRS scores of the 2 groups range from 18.77 to 19.23, suggesting that participants in both groups were experiencing mild to moderate depressive symptoms.

### 3.2. Change in Depression Score after Training

**Figure 2:**
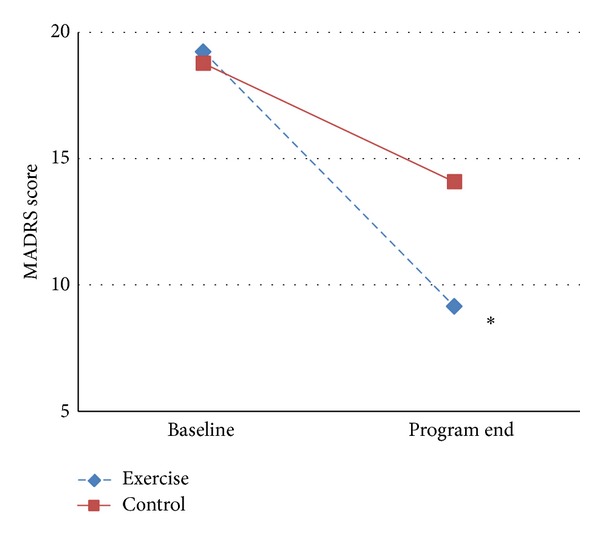
Mean change of MADRS score at baseline and program end. MADRS: Montgomery-Asberg Depression Rating Scale; *P < 0.05 versus control group.

**Figure 3:**
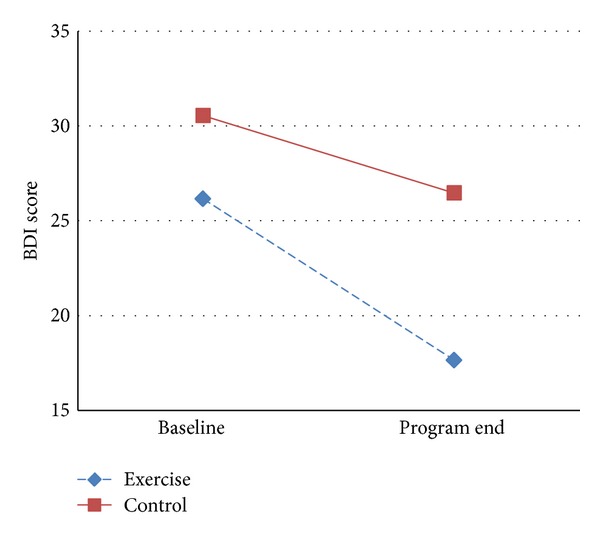
Mean change of BDI score at baseline and program end. BDI: Beck Depression Inventory.

At end of the study, both exercise and control groups showed a significant reduction in the 2 depression scores after 3 weeks of training (Table 2). In the exercise group, after intervention mean score reduced was 10.08 ± 9.4 in MADRS and 8.5 ± 11.36 in BDI; both changes were statistically significant (BDI *P* = 0.001 and MADRS *P* = 0.000). In the control group, a less significant decrease in the depression scores was observed; mean change in MADRS was 4.69 ± 7.33 (*P* = 0.001) and mean change in BDI was 4.08 ± 9.14 (*P* = 0.031) (Table 2). However, when examining the between-group differences in the mean change brought about by treatment effect of aerobic training as compared with control, a significantly greater reduction was reported in MADRS only (*P* = 0.003) (Figure 2). Unlike results in MADRS, the reduction of depressive symptoms when comparing effect of treatment to control was found to be not significant in BDI score (*P* = 0.103) (Figure 3). A clinical response (final scores of 10 or below on the MADRS indicate remission) was observed in 14 (54%) patients in the exercise group but in only 7 (27%) patients in the control group (*P* = 0.007).

### 3.3. Changes in Physical Performance (Handgrip and Flexibility)

After 3 weeks of training, exercise group showed a significant improvement in only one physical domain (sit-and-reach flexibility) (Table 2). In exercise group, after intervention mean sit-and-reach distance increased from 17.22 ± 10.2 cm to 21.62 ± 7.89 cm (*P* = 0.001), while in control group, no significant increase in sit-and-reach distance was observed (change in distance was from 16.48 ± 10.59 to 17.25 ± 11.11; *P* = 0.54). For handgrip strength, both exercise group and control group showed no significant improvement after 3 weeks of training (exercise group: *P* = 0.82; control group: *P* = 0.48).

## 4. Discussion

The results of our study demonstrated that aerobic exercise in addition to pharmacological intervention is effective in leading to a substantial reduction in depressive symptoms among the Chinese population with mild to moderate depression. In fact, 14 (54%) patients in the exercise group but only 7 (27%) patients in the control group had a MADRS score of 10 or below indicating a remission after completion of 3 weeks of program. In addition, the MADRS scores in exercise group were considerably lower than in the control group at the end of the study. Thus, the results of our study suggest that aerobic exercise training is effective in reducing depressive symptoms for patients with mild to moderate depression in the first 3 weeks of hospitalization until antidepressants take effect.

Our findings mirror the results of overseas studies that substantial alleviation of depressive symptoms was revealed in patients with major depressive disorder who underwent supervised aerobic training of moderate to high intensity in a short period of time [27, 28]. However, both previous studies had methodological limitations, including small sample [27, 28] and lack of control [27] in the study design. Our results are in contrast with the findings of two previous randomized controlled trials which failed to show significant differences in depressive scores in older depressed patients after exercise training [29, 30]. This may be due to the different clinical conditions of the studied population and intensity of exercise program that patients in previous trials only exercised three times a week. In our study, subjects received moderate intensity of supervised aerobic training five times a week. Thus, it is suggested that it is necessary to reach a certain threshold of energy expenditure to achieve a significant reduction of depression scores in a short time.

It was no surprise that, in both aerobic exercise and control groups, reduction of depressive symptoms was reported simultaneously in BDI and MADRS scores, meaning that the pharmacological treatment was taking effect and was beyond the “zone of uncertainty” [31], the nonresponse period of antidepressants. Nowadays stringent evidence-based guidelines for treating depressive disorders with antidepressants were adopted by clinicians where antidepressants are a first line treatment for major depression in adults [32]. However under a similar regime of pharmacological treatment, depressive symptoms of our subjects in the aerobic exercise group were more significantly reduced than those in control group, meaning that moderate intensity supervised aerobic exercise training is an effective augmentation to antidepressants and especially valuable during the nonresponse period of antidepressants.

In the present study, the between-group treatment effect of the aerobic exercise training as compared with control was demonstrated in MADRS score but not in BDI; this discrepancy may be due to differences in administrative method and the very nature of the tools and of course sensitivity of the measurements. BDI was developed with a structure which reflects the mood, the inner world, and the value of the patient him/herself, which can be subdivided into the somatic and affective domains. It measures attitudes and cognition which are fairly stable over time among depressed patients and therefore may underestimate the degree of improvement during treatment [33]. In the present study, MADRS was not self-administered but assessed by blinded trained personnel. As commented by Svanborg and Asberg [34], the two scales were equivalent with high intercorrelation (*r* = 0.869) only if both were used as a self-assessment instrument. Since MADRS focused on core depressive symptoms, while BDI was demonstrated to tap more maladaptive personality, subjects self-perception of improvement might therefore be less sensitive to change. Furthermore, among some subgroups, like presence of psychosis, lack of insight, and severe hypochondriasis, self-rating scales were associated with smaller effect sizes, thus rendering a smaller improvement in BDI [35].

In the aerobic exercise group, subjects showed significant improvement in body flexibility (sit-and-reach distance) as compared with those in control. Standard stretching exercise of a total of 10 minutes was given to both groups either in one go (control group) or prior to and after the interval training (aerobic group). Hence the improvement in flexibility would not be due to stretching alone but contributed by the 30-minute aerobic training whose positive effect has already been presented in many studies [36–38] not only on flexibility but also on muscle endurance and feeling of fatigue. Canales et al. [39] reported that, during episodes of depression, individuals with major depressive disorder experience changes in posture and mild dissatisfaction with body image; this negative impact might be counterbalanced by the improvement in body flexibility. Unlike flexibility, no improvement was observed in handgrip strength in neither the exercise nor the control group. It might be because no component of strengthening was included in this study and actually there was no obvious decline in handgrip strength as compared with norm data [40].

Intensity of exercise correlates positively with cognitive flexibility and gives feeling of control and fosters social interaction [41, 42]. In fact, both group and individual training are reported to be equally effective in reducing depression [43, 44]. Therefore, effect of social interaction cannot explain results of the present study particularly when the reduction in depression scores was substantially greater in the exercise group indicating an additional effect of aerobic training on depression.

Although the mechanisms accounted for exercise related improvements in depressive symptoms are not known, numerous biological and psychosocial frameworks have been hypothesized to explicate the antidepressant effects of exercise. Exercise may increase serotonin synthesis and reuptake in the brain [45]. Secondly, exercise training can lead to attenuation of the HPA axis response to stress [46]. Thirdly, regular exercise may increase neurogenesis in the hippocampus, a mechanism that has been related to the action of antidepressants [47].

Public healthcare services in Hong Kong are heavily subsidized up to 84% to 98% by the government [48]. The longer the hospitalization is, the more significant the financial impact will be. Early introduction of supervised aerobic training in in-patient phase can have a synergistic effect with pharmacological intervention to reduce depressive symptoms in a short period of time thus minimizing the impact.

There were a few limitations in this study. First of all, this study did not follow up subjects long enough to examine the long-term effect of aerobic exercise on the symptom reduction if the individuals failed to continue the exercise regime. Secondly, the sample size was relatively small as compared to studies of pharmacological treatment and the number of male participants was small. Above all, we detected a lower sensitivity in the BDI which supported the need for a larger sample size to adjust for this smaller one in effect size.

## 5. Conclusion

There are times when antidepressants have a latency of several weeks until taking effect. During this nonresponse period, both patients and physicians are confronted with a lack of therapeutic options in reducing depressive symptoms in a short period of time. This study demonstrated encouraging results that aerobic exercise training in addition to pharmacological intervention is effective in reducing depressive symptoms and increasing body flexibility among Chinese patients with mild to moderate depression. Early introduction of exercise training in in-patient phase in treating depression can have a synergistic effect with pharmacological intervention in order to ensure the efficacy of rehabilitation.

## Figures and Tables

**Table 1:** Demographics, clinical outcomes of subjects collected, *P* value, and 95% CI of difference between exercise group and control group at baseline.

	Exercise group	Control group	*P* values	95% CI
	(*n* = 26)	(*n* = 26)		
Number of females (%)	17 (65%)	18 (69%)	0.77	−0.3–0.23
Age (years)	43.62 ± 13.3	48.81 ± 11.30	0.14	−12.07–1.68
BMI (kgm^−2^)	22.33 ± 3.31	23.22 ± 4.61	0.43	−3.13–1.34
MADRS	19.23 ± 10.48	18.77 ± 10.14	0.87	−5.28–6.20
BDI	26.15 ± 10.63	30.53 ± 11.67	0.16	−10.60–1.83
Handgrip (kg)	34.10 ± 18.70	28.06 ± 18.12	0.24	−4.21–16.30
Sit-and-reach distance (cm)	17.22 ± 10.20	16.45 ± 10.59	0.79	−5.01–6.57

BMI: body mass index; BDI: Beck Depression Inventory; MADRS: Montgomery-Asberg Depression Rating Scale; CI: confidence interval.

**Table 2:** Means and SD for clinical outcomes by group at baseline and program end.

	Exercise group (*n* = 26)		Control group (*n* = 26)		*P* value (between-group)	95% CI (between-group)
	Baseline	Program end	Baseline	Program end		
MADRS	19.23 ± 10.48	9.15 ± 7.27	18.77 ± 10.14	14.08 ± 9.04		
Mean change	10.08 ± 9.41		4.69 ± 7.33		0.26	−10.08 to −0.69
*P* value	0.000		0.003			

BDI	26.15 ± 10.63	17.65 ± 11.15	30.54 ± 11.67	26.46 ± 15.05		
Mean change	8.50 ± 11.36		4.08 ± 9.14		0.13	−10.18 to 1.32
*P* value	0.001		0.032			

Sit-and-reach distance (cm)	17.22 ± 10.2	21.62 ± 7.89	16.45 ± 10.59	17.25 ± 11.11		
Mean change	4.4 ± 6.13		0.8 ± 4.71		0.02	0.55 to 6.64
*P* value	0.001		0.87			

Handgrip strength (kg)	34.10 ± 18.70	34.41 ± 17.24	28.06 ± 18.12	29.12 ± 16.96		
Mean change	0.31 ± 7.02		1.06 ± 7.51		0.71	−4.8 to 3.3
*P* value	0.82		0.48			

BDI: Beck Depression Inventory; MADRS: Montgomery-Asberg Depression Rating Scale; CI: confidence interval.
